# Worldwide research productivity in the field of psychiatry

**DOI:** 10.1186/s13033-017-0127-5

**Published:** 2017-02-14

**Authors:** Jinghua Zhang, Xiaoou Chen, Xin Gao, Huizeng Yang, Zhong Zhen, Qingwei Li, Yiqun Lin, Xiyan Zhao

**Affiliations:** 1 Department of Psychiatry, Tianjin Anding Hospital (Tianjin City Mental Health Center), Tianjin, China; 2 Graduate School, Tianjin Medical University, Tianjin, China; 3 Department of Psychiatry, First Teaching Hospital of Tianjin University of Traditional Chinese Medicine, Tianjin, China; 4 Guang’anmen Hospital, China Academy of Chinese Medical Sciences, No. 5 BeiXianGe St., XiCheng District, Beijing, 100053 China

**Keywords:** Psychiatry, Productivity, Literature, Citation

## Abstract

**Background:**

The field of psychiatry has seen significant progress in recent years due to worldwide contributions. National productivity, however, in the field of psychiatry is still unclear. In our study, we investigated contributions of individual nations to the field of psychiatry.

**Methods:**

The Web of Science was used to perform a search from 2011 to 2015 on the subject category “psychiatry”. The total number of articles, citations and the per capita numbers were obtained to analyze the contributions of different countries.

**Results:**

In psychiatry journals from 2011 to 2015, 84,760 articles were published worldwide. The most productive world areas were North America, East Asia, Europe and Oceania. The percentage of articles published in high-income countries was 87.77%, middle-income countries published 12.07%, and lower-income published 0.16%. Most articles were published by the United States (32.68%); the United Kingdom was next (8.59%), which was followed by Germany (6.77%), Australia (5.87%), and Canada (4.9%). The country with the highest number of citations (243,394) was the United States. A positive correlation was found between the population/GDP and the number of publications (P < 0.01). Australia ranked the highest when normalized to population size, and the Netherlands and Norway were next. The Netherlands ranked highest, followed by Israel and Australia when adjusted for GDP.

**Conclusions:**

The authorship of most of the psychiatry articles was from high-income countries and few papers came from low-income countries. The most productive country was the United States. However, when normalized to population size and GDP, some European and Oceania countries were most productive.

**Electronic supplementary material:**

The online version of this article (doi:10.1186/s13033-017-0127-5) contains supplementary material, which is available to authorized users.

## Background

The field of psychiatry has grown significantly in recent years. The dramatic progress in the care of psychiatric disorders can be attributed to contributions worldwide. It is obvious that the scientific contributions to psychiatry should not be the same throughout the world since different countries have different healthcare systems, scientific programs and financial supports [1, 2].

An important process in scientific research is publication of research studies and publications are important for the advancement of psychiatry. An indicator of scientific productivity is the number of articles published. Also, analysis of national productivity can be used to identify tendency in scientific publications. The importance of papers is also a measure of scientific productivity [3]. Assessment of national representation has also been carried out in recent years in several medical fields including endocrinology and metabolism [3], rheumatology [4], emergency medicine [5], critical care medicine [6], stroke research [7], tuberculosis [8], infectious disease [9], nutrition and dietetics [10], cardiovascular disease [11], spine surgery [12], surgical oncology [13], foot and ankle research [14], arthroscopy [15], the hand and wrist literature [16], neurosurgery [17], and radiology [18].

In the field of psychiatry, Igoumenou et al. [19] had analyzed the geographic trends of scientific output and the characteristics of citations using the articles published between 2004 and 2009. However, they did not investigate all the countries worldwide contributed to the field of psychiatry. Moreover, the relationship between the research productivity of countries and their population size and economic status was also not analyzed in their study [19]. Therefore, it is necessary to establish the current worldwide research productivity in psychiatry. We performed an analysis to investigate the national research output in various countries worldwide in psychiatry journals from 2011 to 2015.

## Methods

The Journal Citation Reports (JCR) was established by the Institute for Scientific Information [20]. In the JCR subject category “psychiatry” for the year 2015, there were 197 journals listed and these journals were included in our study (Additional file 1).

The Web of Science database was used for a computerized search in July 2016. Web of Science was chosen because it stores citations and other scholastic influence information, it is considered the leading database, and it has been used in similar types of studies [3, 4, 6, 9, 13–16]. We identified articles published in the 197 journals from January 2011 to December 2015. We only included original articles and reviews. We excluded letters, editorial material and corrections. Titles of the journals were used for the search. The country of the correspondence was taken as the source country for all articles.

The number of papers was used as the indicator of quantity for scientific productivity. The quality indicator was the total number of citations. The number of papers from the countries were the primary outcomes of our study. The countries were ranked according to the number of articles produced. The proportions of articles for high income, upper-middle income, lower-middle income, and low income countries were determined according to the income categories of the World Bank [21]. Gross National Income was used for categorization as well, which was high income ($12,746 or more), upper-middle income ($4126–$12,745), lower-middle income ($1046–$4125), and low income ($1045 or less) [21].

Next, we investigated the papers from the most productive countries; that is, those publishing at least 1% of the total number of papers, incorporating the total number, per capita number, total citations (the number of papers multiplied by their citations) and the mean number of citations. The most recent report from the Central Intelligence Agency was used to determine the population size in each country [22]. The list of journals that had articles published from the top five countries was collected and the top 5 journals of these were noted.

The aim of this study was not to investigate the relative contributions among countries, so descriptive statistics; i.e., sums or averages, were calculated to describe the trends in publication. A Spearman’s test was used to determine the statistical significance of the correlations [3, 6, 14–16]. SPSS 19.0 (SPSS Inc., Chicago, IL, USA) was used for the statistical tests. *P* < 0.05 was noted to be statistically significant. StatPlanet software (StatSilk, Sydney, NSW 2089, Australia) was used to create the world map.

## Results

Using the database of the Web of Science, we identified the number of papers published in psychiatry for the years 2011–2015. The total number was 84,760. A total of 14,902 papers were reported in 2011, and 18,744 papers were produced in 2015. This represented a 1.26-fold increase between 2011 and 2015.

**Fig. 1:**
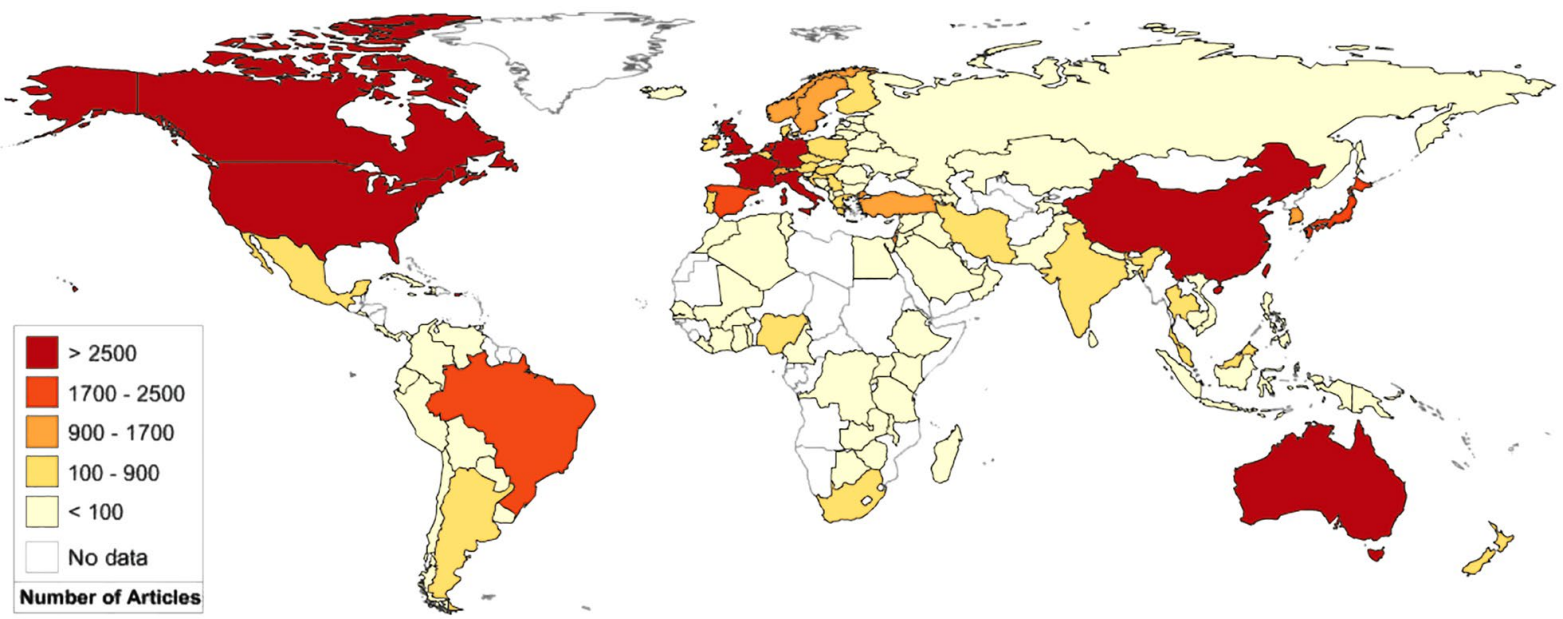
The world map of the worldwide research productivity in the field of psychiatry from 2011 to 2015

**Fig. 2:**
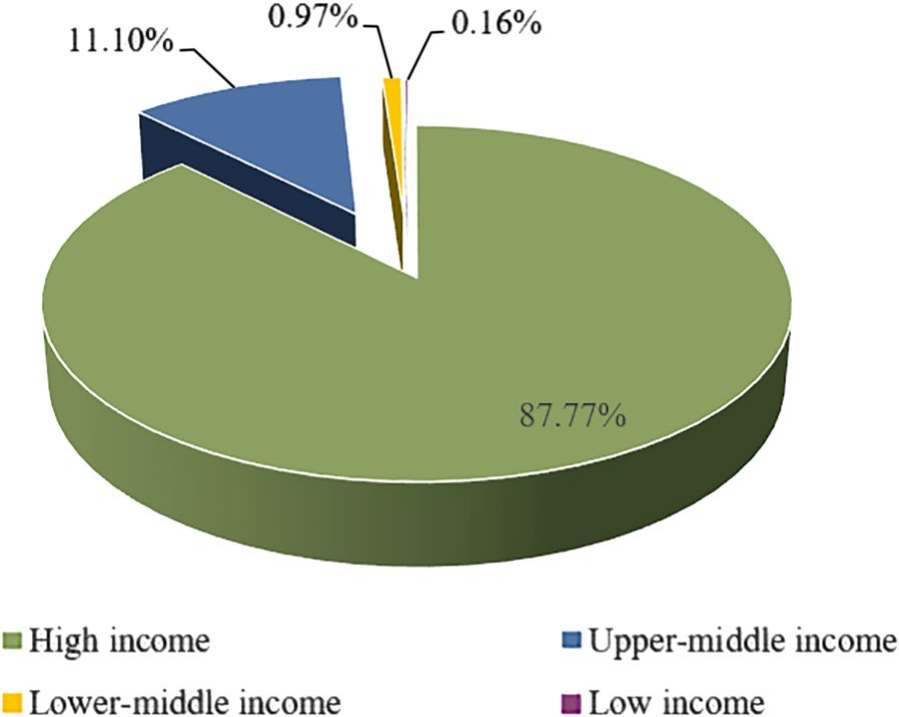
Publications grouped by gross national income from 2011 to 2015

One hundred and twenty-two countries contributed publications to the field of psychiatry. Most articles were published from the United States (27,703/84,760, or 32.68%). This was followed by the United Kingdom (7278/84,760, or 8.59%), Germany (5742/84,760, or 6.77%, Australia (4974/84,760, or 5.87%), and Canada (4156/84,760, or 4.90%). The world map of productive areas is depicted in Fig. 1 and it shows the most productive areas from 2011 to 2015 were North America, East Asia, Europe and Oceania. High income countries had 74,391 publications (87.77%) and upper-middle and lower-middle income countries together had 10,234 publications (12.07%). Only 135 articles (0.16%) were published by low income countries (Fig. 2). The number of papers and population size and GDP were strongly correlated (*P* < 0.01).

Of the total articles (84,760), 75,731 (89.35%) were published by the 18 most productive countries, which produced at 1% of the total papers each (Table 1). A majority of these productive countries were high income countries (15). Upper-middle income countries accounted for the sixth, tenth and thirteenth ranked nations, which were China, Brazil, and Turkey, respectively. Considering the most productive 18 countries, the United States has the most total citations (243,394), the United Kingdom had 71,989 citations, and Germany had 41,004 citations. The United Kingdom had the highest mean number of citations, which was 9.89, and the Netherlands had a mean number of 9.77 and the United States had a mean number of 8.79.

**Table 1:** Publications in the most productive countries from 2011 to 2015

Country	N	%	N per million population	N per 10 billion US $ GDP	Total citation	Mean citation
United States	27,703	32.68	86.87	16.49	243,394	8.79
United Kingdom	7278	8.59	114.18	28.87	71,989	9.89
Germany	5742	6.77	70.89	15.80	41,004	7.14
Australia	4974	5.87	220.99	31.87	37,206	7.48
Canada	4156	4.90	119.31	22.75	32,617	7.85
China	3483	4.11	2.53	3.66	20,670	5.93
Netherlands	3383	3.99	200.45	42.28	33,058	9.77
France	2813	3.32	42.45	10.29	12,824	4.56
Italy	2693	3.18	43.66	13.00	18,706	6.95
Brazil	2216	2.61	10.93	9.87	11,450	5.17
Spain	2144	2.53	44.91	15.78	14,822	6.91
Japan	2062	2.43	16.22	4.21	11,217	5.44
Turkey	1435	1.69	17.58	17.50	3216	2.24
South Korea	1254	1.48	25.57	9.61	6064	4.84
Sweden	1237	1.46	127.21	22.13	9032	7.30
Switzerland	1147	1.35	142.28	17.64	8887	7.75
Israel	1007	1.19	128.74	34.56	6034	5.99
Norway	1004	1.18	195.04	19.59	7249	7.22

*N* number, *GDP* gross domestic product

Australia had the most articles per million population (220.99), followed by the Netherlands (200.45), and Norway (195.04). The Netherlands ranked the highest (42.28), followed by Israel (34.56), and Australia (31.87) after adjustment for GDP. The United States, China, and Japan, which are countries with large economies, ranked relatively low when adjusted by GDP.

Table 2 shows the publication information from the top five countries. *Drug and Alcohol Dependence* was the top journal in the United States, *Psychological Medicine* was the top journal in the United Kingdom, *Nervenarzt* was the top journal in Germany, *Australasian Psychiatry* was the top journal in Australia, and the *Canadian Journal of Psychiatry*-*Revue Canadienne De Psychiatrie* was the top journal in Canada. The *Journal of Affective Disorders* was in the list for 3 of the top 5 countries.

**Table 2:** Ranking journals in the top 5 countries

Rank	United States (27703)	UK (7278)	Germany (5742)	Australia (4974)	Canada (4156)
1	Drug Alcohol Depend (1227)	Psychol Med (345)	Nervenarzt (653)	Australasian Psychiatry (439)	Can J Psychiatry (304)
2	Psychopharmacology (837)	Br J Psychiatry (280)	Fortschr Neurol Psychiatr (309)	Aust N Z J Psychiatry (341)	J Affect Disord (139)
3	Psychiatric Services (790)	J Affect Disord (267)	Psychiatrische Praxis (239)	J Affect Disord (274)	Schizophr Res (131)
4	Neuropsychopharmacology (719)	J Neurol Neurosurg Psychiatry (253)	Eur Arch Psychiatry Clin Neurosci (191)	Psychiatr Psychol Law (182)	Psychiatry Res (123)
5	Schizophr Res (691)	J Psychopharmacol (216)	Prax Kinderpsychol Kinderpsychiatr (179)	Int J Ment Health Nurs (154)	Psychopharmacology (94)

*UK* United Kingdom, *Drug Alcohol Depend* Drug and Alcohol Dependence, *Schizophr Res* Schizophrenia Research, *Psychol Med* Psychological Medicine, *Br J Psychiatry* British Journal of Psychiatry, *J Affect Disord* Journal of Affective Disorders, *J Neurol Neurosurg Psychiatry* Journal of Neurology, Neurosurgery, and Psychiatry, *J Psychopharmacol* Journal of Psychopharmacology, *Fortschr Neurol Psychiatr* Fortschritte Der Neurologie Psychiatrie, *Eur Arch Psychiatry Clin Neurosci* European Archives of Psychiatry and Clinical Neuroscience, *Prax Kinderpsychol Kinderpsychiatr* Praxis Der Kinderpsychologie Und Kinderpsychiatrie, *Aust N Z J Psychiatry* Australian and New Zealand Journal of Psychiatry, *Psychiatr Psychol Law* Psychiatry Psychology and Law, *Int J Ment Health Nurs* International Journal of Mental Health Nursing, *Can J Psychiatry* Canadian Journal of Psychiatry-Revue Canadienne De Psychiatrie, *Psychiatry Res* Psychiatry Research

Table 3 shows the top 5 journals that were used for publication in the five most prolific countries. For the top 5 journals, the United States was most productive. Moreover, the United Kingdom and the United States were within the top 5 countries for all of the top 5 journals. China was in 4 of the 5 top journals. Australia and Canada were in 3 of the top 5 journals.

**Table 3:** Top 5 countries in the top 5 journals

Rank	J Affect Disord (3086)	Psychiatry Res (2621)	Psychopharmacology (1948)	Schizophr Res (1853)	Drug Alcohol Depend (1824)
1	United States (643)	United States (622)	United States (837)	United States (691)	United States (1227)
2	Australia (274)	China (215)	United Kingdom (186)	Canada (131)	Australia (88)
3	United Kingdom (267)	Germany (178)	Netherlands (98)	China (123)	United Kingdom (74)
4	China (242)	United Kingdom (161)	Canada (94)	United Kingdom (116)	Canada (60)
5	Netherlands (168)	Italy (144)	Germany (78)	Australia (94)	China (60)

*J Affect Disord* Journal of Affective Disorders, *Psychiatry Res* Psychiatry Research, *Schizophr Res* Schizophrenia Research, *Drug Alcohol Depend* Drug and Alcohol Dependence

## Discussion

The field of psychiatry has evolved recently, and this can be explained by the great development in science and technology, especially the worldwide scientific contributions of psychiatry researchers. Scientific publications are key indicators of research productivity as well as promoters of the expansion of new knowledge. Worldwide research productivity in several biomedical fields has been evaluated in many recent studies [3–18]. In our study, we evaluated worldwide research productivity in psychiatry from 2011 to 2015.

The results from our study indicated that the United States had more papers than any other country. Therefore, it can be said that the United States plays an important role in psychiatry research. It is well known that the United States has been the most prolific country in many medical fields for several decades. In addition to the field of psychiatry, the United States has been the most productive in many other areas of biomedical research [3, 5, 6, 14–16]. This result is similar with the study from Igoumenou et al. [19]. It indicated that the United States had also been leading the research productivity in psychiatry for many years. However, the other countries, such as Germany and the United Kingdoms, may not keep their ranks when compared with their publication activity in the past years [19].

The United States also has the most citations (243,394) in the field of psychiatry and the second highest mean number of citations (8.79), which suggests that the publications reported by the United States were not only high in number but were also of high quality. Our results indicated that, overall, the United States was the most prolific country in psychiatry.

A “10/90” divide describes the proportions of non-high and high income countries in many medical fields and this can be considered when analyzing the contributions of different countries [3, 5, 6, 23]. A slightly higher proportion, however, was observed for middle income countries in the present study. It may be because of the contributions of China, Turkey, and Brazil, which were productive middle income countries [3]. These countries have been shown to have increasing importance in biomedical research [3, 5, 24, 25]. This is suggestive of the significant development of the societies and economies of these middle-income countries [3, 5]. Rapid economic development in these countries could further improve productivity in psychiatry research and their rank in future studies will be higher. In addition, low research productivity in psychiatry in low income countries was found with only 135 articles identified. A combination of factors may contribute to this, such as medical infrastructures, research characteristics, government policies, and research funding [3, 23].

Australia, the Netherlands, and Norway were more prolific when adjusted by population size. The Netherlands, Israel, and Australia were most prolific with the largest number of papers per capita when normalized to GDP. The most productive countries were nearly all considered as developed countries. Clearly, it may make most sense to normalize, not population size, but by the number of researchers in the countries. It is difficult, though, to determine the number of researchers in psychiatry in each country.

The most popular journals in the United States, United Kingdom, Germany, Australia, and Canada, were *Drug and Alcohol Dependence*, *Psychological Medicine*, *Nervenarzt*, *Australasian Psychiatry* and the *Canadian Journal of Psychiatry*-*Revue Canadienne De Psychiatrie*, respectively. Of note, all of these journals are published in the respective countries. It is possible that more submissions come from countries where the journals are located than from other countries. Also, the United States and the United Kingdom appeared 5 times in the top 5 countries in the top 5 journals. This suggests the impact of these countries in psychiatry researches.

This study had limitations. First, JCR were done to identify journals for this study. Papers reported in non-JCR-cited journals may have contributed to scientific productivity but not included. Second, because only English language journals were incorporated, there may have been a language bias in the journal sources. Third, journals were identified under the “psychiatry” category; however, there may have been articles in the basic science and general internal medicine journals that were not targets of the search. However, the 197 psychiatry journals included in this analysis are likely the most important journals in the field of psychiatry. Fourth, self-citation was not investigated in this study. It may influence the citation index and magnify the importance or popularity of the papers.

## Conclusion

In our study, we evaluated research productivity in the field of psychiatry of various nations throughout the world over a 5-year recent period. Our results showed that most papers came from high income countries and there were fewer publications from low income countries. The most prolific country in psychiatry was the United States. Some European and Oceania countries, however, were most prolific when adjusted by population size and GDP.

## Additional file

**Additional file 1.** List of considered journals under the topic heading “psychiatry” in the Web of Science.

## Abbreviations

GDP: gross domestic product
JCR: Journal Citation Reports
