# Supplementary material for: Worldwide research productivity in the field of psychiatry
Source: Int J Ment Health Syst. 2017 Feb 14;11:20. doi: 10.1186/s13033-017-0127-5 (PMC5310092; doi:10.1186/s13033-017-0127-5)
Supplement: Supplementary file 1 — Additional file 1. List of considered journals under the topic heading “psychiatry” in the Web of Science. [file 13033_2017_127_MOESM1_ESM.docx]

Supplement 1. List of considered journals under the topic heading “Psychiatry” on Web of Science.

| Journal |
| --- |
| *Academic Psychiatry* |
| *Acta Neuropsychiatrica* |
| *Acta Psychiatrica Scandinavica* |
| *Actas Espanolas de Psiquiatria* |
| *Addiction* |
| *Aging & Mental Health* |
| *American Journal of Geriatric Psychiatry* |
| *American Journal of Medical Genetics Part B-Neuropsychiatric Genetics* |
| *American Journal of Orthopsychiatry* |
| *American Journal of Psychiatry* |
| *Anadolu Psikiyatri Dergisi-Anatolian Journal of Psychiatry* |
| *Annales Medico-Psychologiques* |
| *Annals of Clinical Psychiatry* |
| *Annals of General Psychiatry* |
| *Anxiety Stress and Coping* |
| *Archives of Clinical Psychiatry* |
| *Archives of Psychiatric Nursing* |
| *Archives of Suicide Research* |
| *Archives of Womens Mental Health* |
| *Arquivos de Neuro-Psiquiatria* |
| *Asia-Pacific Psychiatry* |
| *Australasian Psychiatry* |
| *Australian and New Zealand Journal of Psychiatry* |
| *Behavioral Medicine* |
| *Behavioral Sleep Medicine* |
| *Biological Psychiatry* |
| *Bipolar Disorders* |
| *BMC Psychiatry* |
| *Body Image* |
| *British Journal of Psychiatry* |
| *Bulletin of The Menninger Clinic* |
| *Canadian Journal of Psychiatry-Revue Canadienne de Psychiatrie* |
| *Child and Adolescent Mental Health* |
| *Child and Adolescent Psychiatric Clinics of North America* |
| *Child and Adolescent Psychiatry and Mental Health* |
| *Child Psychiatry & Human Development* |
| *Clinical Case Studies* |
| *Clinical Child Psychology and Psychiatry* |
| *Clinical EEG and Neuroscience* |
| *Clinical Gerontologist* |
| *CNS Drugs* |
| *CNS Spectrums* |
| *Cognitive Neuropsychiatry* |
| *Community Mental Health Journal* |
| *Comprehensive Psychiatry* |
| *Contemporary Psychoanalysis* |
| *Criminal Behaviour and Mental Health* |
| *Crisis-The Journal of Crisis Intervention and Suicide Prevention* |
| *Culture Medicine and Psychiatry* |
| *Current Opinion in Psychiatry* |
| *Current Psychiatry Reports* |
| *Dementia and Geriatric Cognitive Disorders* |
| *Depression and Anxiety* |
| *Drug and Alcohol Dependence* |
| *Early Intervention in Psychiatry* |
| *Eating and Weight Disorders-Studies on Anorexia Bulimia and Obesity* |
| *Eating Behaviors* |
| *Eating Disorders* |
| *Encephale-revue De Psychiatrie Clinique Biologique Et Therapeutique* |
| *Epidemiology and Psychiatric Sciences* |
| *Epilepsy & Behavior* |
| *European Addiction Research* |
| *European Archives of Psychiatry and Clinical Neuroscience* |
| *European Child & Adolescent Psychiatry* |
| *European Journal of Psychiatry* |
| *European Journal of Psychotraumatology* |
| *European Neuropsychopharmacology* |
| *European Psychiatry* |
| *Evolution Psychiatrique* |
| *Experimental and Clinical Psychopharmacology* |
| *Fortschritte der Neurologie Psychiatrie* |
| *General Hospital Psychiatry* |
| *Geriatrie et Psychologie Neuropsychiatrie de Vieillissement* |
| *Harvard Review of Psychiatry* |
| *History of Psychiatry* |
| *Human Psychopharmacology-Clinical and Experimental* |
| *International Clinical Psychopharmacology* |
| *International Journal of Clinical and Experimental Hypnosis* |
| *International Journal of Cognitive Therapy* |
| *International Journal of Eating Disorders* |
| *International Journal of Forensic Mental Health* |
| *International Journal of Geriatric Psychiatry* |
| *International Journal of Law and Psychiatry* |
| *International Journal of Mental Health and Addiction* |
| *International Journal of Mental Health Nursing* |
| *International Journal of Mental Health Promotion* |
| *International Journal of Mental Health Systems* |
| *International Journal of Methods in Psychiatric Research* |
| *International Journal of Neuropsychopharmacology* |
| *International Journal of Psychiatry in Clinical Practice* |
| *International Journal of Psychiatry in Medicine* |
| *International Journal of Social Psychiatry* |
| *International Psychogeriatrics* |
| *International Review of Psychiatry* |
| *Issues in Mental Health Nursing* |
| *JAMA Psychiatry* |
| *Journal of Affective Disorders* |
| *Journal of Aggression Maltreatment & Trauma* |
| *Journal of Anxiety Disorders* |
| *Journal of Attention Disorders* |
| *Journal of Behavior Therapy and Experimental Psychiatry* |
| *Journal of Behavioral Addictions* |
| *Journal of Child and Adolescent Psychopharmacology* |
| *Journal of Child and Family Studies* |
| *Journal of Child Psychology and Psychiatry* |
| *Journal of Clinical Psychiatry* |
| *Journal of Clinical Psychopharmacology* |
| *Journal of Dual Diagnosis* |
| *Journal of ECT* |
| *Journal of Forensic Psychiatry & Psychology* |
| *Journal of Geriatric Psychiatry and Neurology* |
| *Journal of Mental Health Policy and Economics* |
| *Journal of Mental Health Research in Intellectual Disabilities* |
| *Journal of Nervous and Mental Disease* |
| *Journal of Neurology Neurosurgery and Psychiatry* |
| *Journal of Neuropsychiatry and Clinical Neurosciences* |
| *Journal of Obsessive-Compulsive and Related Disorders* |
| *Journal of Personality Disorders* |
| *Journal of Psychiatric and Mental Health Nursing* |
| *Journal of Psychiatric Practice* |
| *Journal of Psychiatric Research* |
| *Journal of Psychiatry & Neuroscience* |
| *Journal of Psychopharmacology* |
| *Journal of Psychosomatic Obstetrics & Gynecology* |
| *Journal of Psychosomatic Research* |
| *Journal of the American Academy of Child and Adolescent Psychiatry* |
| *Journal of the American Academy of Psychiatry and the Law* |
| *Journal of the American Psychiatric Nurses Association* |
| *Journal of the American Psychoanalytic Association* |
| *Journal of the International Neuropsychological Society* |
| *Journal of Trauma & Dissociation* |
| *Journal of Traumatic Stress* |
| *Klinik Psikofarmakoloji Bulteni-Bulletin of Clinical Psychopharmacology* |
| *Lancet Psychiatry* |
| *Mental Health and Physical Activity* |
| *Mindfulness* |
| *Molecular Psychiatry* |
| *Nervenarzt* |
| *Neurocase* |
| *Neuropsychiatric Disease and Treatment* |
| *Neuropsychiatry* |
| *Neuropsychobiology* |
| *Neuropsychopharmacology* |
| *Nordic Journal of Psychiatry* |
| *Personality and Mental Health* |
| *Perspectives in Psychiatric Care* |
| *Pharmacopsychiatry* |
| *Praxis der Kinderpsychologie und Kinderpsychiatrie* |
| *Progress in Neuro-Psychopharmacology & Biological Psychiatry* |
| *Psychiatria Danubina* |
| *Psychiatria Polska* |
| *Psychiatric Annals* |
| *Psychiatric Clinics of North America* |
| *Psychiatric Quarterly* |
| *Psychiatric Rehabilitation Journal* |
| *Psychiatric Services* |
| *Psychiatrie De L Enfant* |
| *Psychiatrische Praxis* |
| *Psychiatry and Clinical Neurosciences* |
| *Psychiatry Investigation* |
| *Psychiatry Psychology and Law* |
| *Psychiatry Research* |
| *Psychiatry Research-Neuroimaging* |
| *Psychiatry-Interpersonal and Biological Processes* |
| *Psychogeriatrics* |
| *Psychological Medicine* |
| *Psychological Trauma-Theory Research Practice and Policy* |
| *Psychology and Psychotherapy-Theory Research and Practice* |
| *Psychoneuroendocrinology* |
| *Psychopathology* |
| *Psychopharmacology* |
| *Psychosis-Psychological Social and Integrative Approaches* |
| *Psychosomatic Medicine* |
| *Psychosomatics* |
| *Psychotherapy and Psychosomatics* |
| *Recht & Psychiatrie* |
| *Research in Autism Spectrum Disorders* |
| *Revista Brasileira de Psiquiatria* |
| *Revista de Psiquiatria y Salud Mental* |
| *Rivista di Psichiatria* |
| *Salud Mental* |
| *Schizophrenia Bulletin* |
| *Schizophrenia Research* |
| *Social Psychiatry and Psychiatric Epidemiology* |
| *South African Journal of Psychiatry* |
| *Stress and Health* |
| *Substance Use & Misuse* |
| *Suchttherapie* |
| *Suicide and Life-Threatening Behavior* |
| *Transcultural Psychiatry* |
| *Translational Psychiatry* |
| *Turk Psikiyatri Dergisi* |
| *Verhaltenstherapie* |
| *World Journal of Biological Psychiatry* |
| *World Psychiatry* |
| *Zeitschrift Fur Kinder-und Jugendpsychiatrie und Psychotherapie* |
| *Zeitschrift fur Psychosomatische Medizin und Psychotherapie* |
